# Intravenous immunoglobulin and Alzheimer’s disease: what now?

**DOI:** 10.1186/1742-2094-10-70

**Published:** 2013-06-05

**Authors:** David A Loeffler

**Affiliations:** 1 Department of Neurology Research, William Beaumont Hospital Research Institute, Beaumont Health System, 3811 West Thirteen Mile Road, Royal Oak, MI 48073, USA

**Keywords:** Aβ, Alzheimer’s disease, Antibodies, Immunotherapy, Inflammation, Intravenous immunoglobulin, Mild cognitive impairment, RAGE, Tau

## Abstract

Intravenous immunoglobulin (IVIG) products are prepared from purified plasma immunoglobulins from large numbers of healthy donors. Pilot studies with the IVIG preparations Octagam and Gammagard in individuals with mild-to-moderate Alzheimer’s disease (AD) suggested stabilization of cognitive functioning in these patients, and a phase II trial with Gammagard reported similar findings. However, subsequent reports from Octagam’s phase II trial and Gammagard’s phase III trial found no evidence for slowing of AD progression. Although these recent disappointing results have reduced enthusiasm for IVIG as a possible treatment for AD, it is premature to draw final conclusions; a phase III AD trial with the IVIG product Flebogamma is still in progress. IVIG was the first attempt to use multiple antibodies to treat AD. This approach should be preferable to administration of single monoclonal antibodies in view of the multiple processes that are thought to contribute to AD neuropathology. Development of “AD-specific” preparations with higher concentrations of selected human antibodies and perhaps modified in other ways (such as increasing their anti-inflammatory effects and/or ability to cross the blood–brain barrier) should be considered. Such preparations, if generated with recombinant technology, could overcome the problems of high cost and limited supplies, which have been major concerns relating to the possible widespread use of IVIG in AD patients. This review summarizes the recent AD IVIG trials and discusses the major issues relating to possible use of IVIG for treating AD, as well as the critical questions which remain.

## Introduction

The severity of the Alzheimer’s disease (AD) problem in the US is indicated by recent statistics from the Alzheimer’s Association [1]. Approximately 5.4 million Americans are currently diagnosed with AD, including one in eight individuals aged 65 and older. By 2025 the number of Americans aged 65 and older with AD is expected to reach 6.7 million. Payments for health care, long-term care, and hospice for AD patients, which are currently estimated at $200 billion, are expected to be $1.1 trillion (in 2012 dollars) by 2050.

Intravenous immunoglobulin (IVIG) products are being investigated as potential agents for treatment or prevention of AD. IVIG is prepared from plasma immunoglobulins from large numbers (**>**10,000) of healthy donors. It is used to treat a range of autoimmune, infectious, and idiopathic disorders [2]. The IVIG product Octagam (Octapharma) was shown by Dodel et al. in 2002 to contain antibodies to Aβ, suggesting that IVIG might be useful for treatment of AD [3]. This provided the rationale for IVIG pilot studies in AD patients. The results of these studies were encouraging [4,5], leading to phase II AD trials with these products. Positive results were reported for one of the two IVIG products [6,7] but not the other one [8]. More recently, negative results were reported in a phase III IVIG study [9]. An additional phase III AD trial with another IVIG product is in progress.

As of this writing (May 2013) it is unclear whether any IVIG products will offer a breakthrough for treatment of AD. Because these preparations differ in their concentrations of antibodies to Aβ [10-12] and tau protein [13], there may be differences between them with regard to their efficacies in AD. This purpose of this review is to summarize the IVIG AD trials conducted to date, the issues raised by the potential use of IVIG for treatment of AD, and critical questions which remain.

### Clinical trials with IVIG in AD patients

IVIG treatment of AD patients was first reported in a pilot study in 2004 [4]. Five patients with mild to moderate AD [Mini Mental State Examination (MMSE) mean score 19.4] received Octagam (Octapharma; dose = 0.4 g/kg) on 3 successive days, every 4 weeks for 6 months. MMSE scores improved slightly in four of the AD patients and were unchanged in the fifth one, while their Alzheimer's Disease Assessment Scale-Cognitive sub-scale (ADAS-cog) scores decreased, suggesting increased cognitive functioning, in four patients and did not change in the fifth one. In 2009 results were published [5] from a pilot study in which eight AD patients (mean MMSE score 23.5) were administered Gammagard S/D (Baxter Healthcare). After 6 months of treatment the mean MMSE score increased to 26.0, reflecting increased scores for six patients and no change in scores for two patients. After a 3-month washout period, the mean MMSE score returned to baseline (23.9). Following an additional 9 months of treatment, MMSE scores were essentially unchanged (mean 24.0).

Before publishing these results, in 2006 Baxter began a double-blind Phase II AD trial with Gammagard. Improved outcomes were noted in the Gammagard-treated subjects compared to those initially treated with placebo at 3, 6, and 9 months [6]. The four patients who received Gammagard at 0.4 g/kg every 2 weeks for 36 months showed no evidence of further cognitive or memory decline [7].

The results of a double-blind, placebo-controlled, 24-week phase II AD trial with Octagam were published in January 2013 [8]. Octagam had no apparent effects on cognitive or functional scores in the AD patients. No increase was found for plasma Aβ1-40; this had been reported in the pilot studies and suggested that IVIG products might increase efflux of Aβ from the brain. The only positive finding reported in this study, less reduction in glucose metabolism in some brain regions in the Octagam-treated individuals, was of uncertain significance.

In May 2013, the results of a placebo-controlled phase III AD trial with Gammagard were announced [9]. Three hundred ninety patients had been treated every 2 weeks for 18 months with 200 mg/kg Gammagard, 400 mg/kg Gammagard, or placebo. No significant differences were found for the rate of cognitive decline between the Gammagard-treated group and placebo group.

Two AD-related IVIG trials are still in progress. Flebogamma (Grifols Biologicals) is being evaluated, together with albumin, in an AD phase III trial, and NewGam (Octapharma) is being investigated by Sutter Health in a phase II trial to determine its effects in patients with amnestic mild cognitive impairment (MCI) and its influence on the risk for these patients to develop AD. A possible reason for the failures in the most recent IVIG trials is that by the time AD’s clinical features become evident, its pathology, including extensive neuronal loss, is already wellestablished. The trial with MCI patients should provide an indication of whether earlier IVIG treatment may be beneficial. The findings of a retrospective study [14], that individuals 65 years of age and older who had received IVIG had a significantly reduced risk for developing AD and related disorders compared to subjects not receiving IVIG support this possibility.

## Considerations relating to treatment of AD with IVIG products

### Economic considerations

Approximately 22 million plasma donations were collected in the US in 2009. Processing of a plasma sample into IVIG requires about 9 months. The use of IVIG for treatment of AD could pose a threat to its supplies. Solutions that have been suggested to this problem include new manufacturing processes, use of recombinant technology to produce IVIG, or administration of specific antibodies in place of IVIG [15]. Because of problems with obtaining adequate amounts of IVIG, some hospitals require consideration of alternatives to IVIG for treatment of patients with neurological disorders [16].

IVIG treatment is expensive, although its actual cost varies with the dose, frequency of administration, and home vs. hospital infusion. The Medicare Modernization Act of 2003 fixed Medicare’s IVIG reimbursement rate to doctors and hospitals at the average sale price for IVIG plus 6%. Although the federal “340B drug discount” program mandates discounted IVIG prices for hospitals that provide high volumes of care to low-income patients [17], some hospitals have been unable to buy IVIG at these prices [18].

### Potential side effects of IVIG

#### Thromboembolic events

IVIG can lead to thromboemboli because it increases serum viscosity, resulting in reduced blood flow [19]. Pre-existing vascular disease and immobility, additional factors that increase the risk for thromboembolic events [20], are often present in elderly individuals, including those with AD [21,22]. In patients with neuropathies that received IVIG, immobility was found to be an independent predictor for the development of thromboemboli [23]. Vascular disease and immobility in AD patients could therefore increase the risk of these individuals for IVIG-induced thromboembolic events.

#### Renal problems

Renal dysfunction, acute renal failure, and osmotic nephrosis can result from IVIG treatment in patients with predisposing conditions such as renal insufficiency, diabetes mellitus, age > 65, volume depletion, sepsis, or receiving nephrotoxic drugs [24,25]. This is particularly true for IVIG products that use sucrose as a stabilizing agent. The prevalence of chronic renal disease increases with age, peaking at nearly 24% at ages 70–74 [26], so elderly individuals are likely to have an increased risk for IVIG-induced kidney problems because of pre-existing renal disease. AD patients with impaired kidney function may therefore not be candidates for IVIG treatment.

#### Hematological deficits

Decreases in WBCs, RBCs, and platelets have been reported after IVIG treatment [27-30]. The mechanisms responsible for these alterations are unclear. IVIG can stimulate platelet aggregation [31], which could reduce platelet counts. Binding of high-molecular-weight IgG complexes in IVIG to RBCs could decrease hematocrit levels in IVIG-treated patients by increasing RBC sequestration [32]. Binding of neutrophils to the vascular wall and migration of these cells into a storage pool has been suggested as a mechanism for IVIG-induced neutropenia [33]. Similar to the situation with regard to renal problems, individuals with AD who have hematological abnormalities may not be able to receive IVIG.

### Potential mechanisms of action of IVIG in AD

IVIG products are thought to contain the full range of antibodies present in the human repertoire, estimated at 10^9^[15]. IVIG’s mechanisms of action in different disorders are generally poorly understood. It contains several antibodies that have the potential to reduce AD-type pathology, but whether these antibodies can actually do so is unclear.

### Anti-Aβ effects

IVIG products contain antibodies to Aβ oligomers and fibrils [34] and perhaps also to monomeric Aβ [11,35]. These drugs differ in their levels of anti-Aβ antibodies [10-12]. IVIG has been shown *in vitro* to disaggregate preformed Aβ fibrils, promote Aβ phagocytic removal [36], protect against Aβ neurotoxicity [35,37], and prevent formation of Aβ soluble oligomers [11]. But studies in mouse models of AD have produced conflicting results as to whether IVIG products can reduce brain Aβ. Magga et al. [38] found that Gammagard promoted microglial-mediated clearance of Aβ in experiments with brain sections from APP/PS1 mice and reduced *in vitro* Aβ fibril formation, but the latter effect was not specific for its anti-Aβ antibodies. Dodel et al. [37] treated APP695 double mutant mice with purified anti-Aβ antibodies from Octagam for 4 weeks beginning at 3 or 12 months of age. Reduced plaque counts were found in the younger mice but not in the older mice. Puli et al. [39] treated TgApdE9 mice with Gammagard beginning at 4 months of age, for 3 or 8 months. In the 3-month-treated group, there were no effects on hippocampal plaque counts or brain Aβ. After 8 months, there were still no differences in plaque counts between treatment and control groups. Surprisingly, soluble Aβ levels in hippocampus were increased in treated mice.

IVIG’s antibodies recognize multiple sites on conformational Aβ epitopes, and its main binding to Aβ is reportedly to Aβ25-40 [12,37]. This differs from the monoclonal anti-Aβ antibodies that have been used in clinical trials, Bapineuzumab and Solanezumab, which recognize only one epitope in linear Aβ and bind to Aβ1-5 and Aβ13-28, respectively [40]. A recent review [41] suggested that using the IVIG polyclonal antibody approach in an effort to deplete the spectrum of aggregated Aβ species might be more promising than using monoclonal antibodies targeting a single Aβ species.

### Anti-inflammatory effects

IVIG can inhibit complement activation [42], neutralize inflammatory cytokines [43], and modulate chemokine expression [44] and regulatory T cell subsets [45]. However, its main anti-inflammatory effects are thought to be due to its IgG Fc fragments [46,47]. IVIG may not have reduced brain inflammation in the AD clinical trials because high doses (1–2 g/kg) are required for it to exert these effects [48]. Ravetch and colleagues investigated the mechanism by which IVIG’s Fc fragment produces anti-inflammatory effects. The constant domain of IgG’s Fc contains a glycan (a core heptapolysaccharide containing N-acetylglucosamine and mannose at Asn^297^) [49]. In serum antibodies, this includes a terminal sialic acid, which is responsible for Fc’s anti-inflammatory effects [50]. This form of the carbohydrate is present in only 1-3% of IVIG’s IgG molecules, which may explain why high-dose IVIG is required to produce anti-inflammatory effects [51].

### Possible anti-tau effects

Cognitive deficits in AD strongly correlate with neurofibrillary tangle (NFT) density and distribution [52,53]. Aggregation and hyperphosphorylation of tau are required to produce NFTs. Studies in transgenic mouse models of tauopathy found that administration of antibodies to phosphorylated tau reduced tau pathology [54,55], so if IVIG contains such antibodies, they might be beneficial for treatment of AD. We recently reported the presence of antibodies to recombinant human tau, which is unphosphorylated, in IVIG products [13]. The significance of these antibodies to “normal” tau (i.e., whether these antibodies can reduce tau oligomer formation or inhibit its neurotoxicity) and whether IVIG also contains antibodies to aggregated and hyperphosphorylated tau are unknown.

### Alteration of Aβ passage in and out of the brain

Advanced glycation endproducts (AGEs) form when reducing sugars react with amino groups in proteins, lipids, and nucleic acids [56]. The receptor for AGEs (RAGE) is present on the blood–brain barrier (BBB) [57], where it transports Aβ into the brain [58]. Anti-RAGE antibodies have been reported in IVIG [59]. These antibodies could reduce Aβ influx into the brain.

Low-density lipoprotein receptor-related protein-1 (LRP1) is the main receptor for transporting Aβ out of the brain [60]. LRP-1 expression on the BBB is reduced in AD [61]. Soluble LRP (sLRP) has also been reported in IVIG [59]. sLRP binds 70–90% of plasma Aβ, preventing it from entering the brain [62], so increasing peripheral blood sLRP levels through IVIG treatment might help to lower brain Aβ levels.

### Non-antibody-mediated effects

IVIG contains other non-antibody proteins in addition to sLRP, which could influence its actions in AD in ways that are not clear. Interferon-γ, an inflammatory cytokine that also has some anti-inflammatory actions [63], is present in IVIG [64]. Soluble human leukocyte antigen (HLA) class I and II molecules are present in some IVIG products, as are their “physiological ligands,” CD4 and CD8 [65,66]. Soluble CD4 in IVIG might interfere with HLA class II molecules on antigen-presenting cells, competing with HLA-class II-restricted T cells and possibly causing immunosuppression [66,67]. Transforming growth factor (TGF)-β1 and TGF-β2 are also present in IVIG [68].

TGF-β1 is increased in AD brain, where it is associated with plaques [69], but it also may promote Aβ clearance [70], so its significance in AD is unclear.

### Might IVIG products differ with respect to their effects in AD?

Differences have been reported between IVIG products for the concentrations of some of their antibodies and their biological activities [71-75]. These may be due to differences in manufacturing practices and/or the antigenic exposure of the plasma donors. With regard to AD, differences between IVIG preparations have been found for anti-Aβ [10-12] and anti-tau antibodies [13], as mentioned earlier. Determination of whether IVIG products differ in their ability to slow AD’s progression will require comparative studies, as have been done for Kawasaki disease [76] and primary immune deficiency [77].

### How might the IVIG approach to AD treatment be improved?

Production of an “AD-specific” human immunoglobulin preparation would allow selected antibodies to be administered in higher concentrations than is possible with unfractionated IVIG. There are precedents for this. Polyclonal anti-D, a type of IVIG consisting of plasma from RhD-negative donors immunized to the D antigen [78], has been used to treat idiopathic thrombocytopenic purpura [79,80], and Shoenfeld and colleagues [81] have generated specific IVIG (sIVIG) preparations for a number of immune-mediated conditions including lupus and antiphospholipid syndrome. These antibodies could be produced with recombinant technology to avoid potential supply difficulties. A second possibility could be to administer multiple “humanized” mouse monoclonal antibodies with antigenic specificities similar to those of the antibodies in the AD-specific human IgG preparations. Single chain antibody fragments, which have been suggested as alternatives to monoclonal antibodies for AD treatment [82], should have better BBB penetration than intact IgG molecules. Still another alternative could be administration of recombinant sialylated Fc fragments, as suggested by Bayry et al. [15] for autoimmune diseases; as discussed above, this modification increases Fc’s anti-inflammatory properties [49,50]. Sialic acid-enriched immunoglobulins have been obtained from IVIG by lectin fractionation [49,83].

### Critical questions remaining

The most important question relating to AD treatment with IVIG is clearly whether any IVIG products will consistently slow clinical progression of this disorder. Questions that, if addressed, could lead to improved efficacy for IVIG in the treatment of AD include the following:

1. Regarding the antibodies in IVIG that have been identified against AD pathological proteins: what are their effects in mouse transgenic models of AD that develop both plaques and NFTs? (Although the complement membrane attack complex, C5b-9, is present on plaques and neurofibrillary tangles in the AD brain [84,85], the currently available mouse transgenic models of AD do not demonstrate this late-stage complement activation [86], so the effects of IVIG’s anti-complement antibodies on this process cannot presently be studied in animal models of AD.)

2. What other AD-related antibodies are present in IVIG? (Does IVIG have antibodies against aggregated or hyperphosphorylated tau or antibodies that have antioxidant effects? Do its anti-Fas antibodies [72] have any relevance for AD treatment?)

3. Do IVIG’s anti-idiotypic antibodies influence its effects in AD? These antibodies are thought to be responsible for IVIG’s benefits in autoimmune disorders [87], but their effects in AD are unknown.

4. Are IVIG’s effects in AD similar between apoE4 carriers and non-carriers? (In the phase III Gammagard trial [9], it was suggested that the 400 mg/kg dose may have been of some benefit to apoE4^+^ AD patients).

5. Does IVIG treatment of AD patients increase their risk for microhemorrhage? (In the phase II Octagam study, 14% of the Octagam-treated patients had microhemorrhages vs. none in the placebo group [8]).

## Conclusions

The IVIG trials reported to date in AD patients have produced conflicting findings. Because the most recent trials produced negative results, enthusiasm for IVIG as a treatment for AD has been reduced, although hope remains that the phase III trial with Flebogamma will produce positive results. Multiple antibody therapy for AD, as typified by IVIG, should have advantages over administration of individual monoclonal antibodies. To identify which antibodies should be included in an AD-specific IVIG preparation, more must be known about the range of anti-AD antibodies in IVIG and their effects on AD pathology in animal models. Such an AD-specific IVIG preparation, modified to increase the concentration, anti-inflammatory activity, and perhaps the brain penetration of its antibodies, might be more beneficial to AD patients than treatment with unfractionated IVIG.

## Competing interests

The author declares that he has no competing interests.

## Authors’ contributions

DAL wrote the manuscript.

## References

[B1] A's A Alzheimer's disease facts and figures Alzheimers Dement 2012 2012 8 131 168 10.1016/j.jalz.2012.02.001 22404854

[B2] Scheinfeld NS Intravenous Immunoglobulinhttp://emedicine.medscape.com/article/210367-overview

[B3] Dodel R Hampel H Depboylu C Lin S Gao F Schock S Jäckel S Wei X Buerger K Höft C Hemmer B Möller HJ Farlow M Oertel WH Sommer N Du Y Human antibodies against amyloid beta peptide: a potential treatment for Alzheimer's disease Ann Neurol 2002 52 253 256 10.1002/ana.10253 12210803

[B4] Dodel RC Du Y Depboylu C Hampel H Frölich L Haag A Hemmeter U Paulsen S Teipel SJ Brettschneider S Spottke A Nölker C Möller HJ Wei X Farlow M Sommer N Oertel WH Intravenous immunoglobulins containing antibodies against beta-amyloid for the treatment of Alzheimer's disease J Neurol Neurosurg Psychiatry 2004 75 1472 1474 10.1136/jnnp.2003.033399 15377700 PMC1738770

[B5] Relkin NR Szabo P Adamiak B Burgut T Monthe C Lent RW Younkin S Younkin L Schiff R Weksler ME 18-Month study of intravenous immunoglobulin for treatment of mild Alzheimer disease Neurobiol Aging 2009 30 1728 1736 10.1016/j.neurobiolaging.2007.12.021 18294736

[B6] Science Daily Results Of 9-Month Phase II Study Of Gammagard Intravenous Immunoglobulinhttp://www.sciencedaily.com/releases/2008/07/080730175522.htm

[B7] Medpage Today IVIG Stops Alzheimer's in Its Tracks http://www.medpagetoday.com/MeetingCoverage/AAIC/33780

[B8] Dodel R Rominger A Bartenstein P Barkhof F Blennow K Förster S Winter Y Bach JP Popp J Alferink J Wiltfang J Buerger K Otto M Antuono P Jacoby M Richter R Stevens J Melamed I Goldstein J Haag S Wietek S Farlow M Jessen F Intravenous immunoglobulin for treatment of mild-to-moderate Alzheimer's disease: a phase 2, randomised, double-blind, placebo-controlled, dose-finding trial Lancet Neurol 2013 12 233 243 10.1016/S1474-4422(13)70014-0 23375965 PMC4986921

[B9] Medpage Today Alzheimer’s Disease: IVIG Fails in Trial http://www.medpagetoday.com/Neurology/AlzheimersDisease/3893923857923

[B10] Safavi A Langevin M Vandeberg P Novokhatny V Scuderi P Mohn G Petteway S Comparison of several human immunoglobulin products for anti-Aβ1–42 titer, 10th International Conference on Alzheimer's Disease and Related Disorders 2006 Madrid, Spain: International Conference on Alzheimer’s Disease

[B11] Klaver AC Finke JM Digambaranath J Balasubramaniam M Loeffler DA Antibody concentrations to Abeta1-42 monomer and soluble oligomers in untreated and antibody-antigen-dissociated intravenous immunoglobulin preparations Int Immunopharmacol 2010 10 115 119 10.1016/j.intimp.2009.10.005 19840873

[B12] Balakrishnan K Andrei-Selmer LC Selmer T Bacher M Dodel R Comparison of intravenous immunoglobulins for naturally occurring autoantibodies against amyloid-beta J Alzheimers Dis 2010 20 135 143 20164596 10.3233/JAD-2010-1353

[B13] Smith LM Coffey MP Klaver AC Loeffler DA Intravenous immunoglobulin products contain specific antibodies to recombinant human tau protein Int Immunopharmacol 2013 16 424 428 [Epub ahead of print] 10.1016/j.intimp.2013.04.034 23669333

[B14] Fillit H Hess G Hill J Bonnet P Toso C IV immunoglobulin is associated with a reduced risk of Alzheimer disease and related disorders Neurology 2009 73 180 185 10.1212/WNL.0b013e3181ae7aaf 19620605

[B15] Bayry J Kazatchkine MD Kaveri SV Shortage of human intravenous immunoglobulin–reasons and possible solutions Nat Clin Pract Neurol 2007 3 120 121 17342189 10.1038/ncpneuro0429

[B16] Winters JL Brown D Hazard E Chainani A Andrzejewski C Jr Cost-minimization analysis of the direct costs of TPE and IVIg in the treatment of Guillain-Barré syndrome BMC Health Serv Res 2011 11 101 10.1186/1472-6963-11-101 21575219 PMC3121582

[B17] Public Hospital Pharmacy Coalition An Overview of The Section 340B Drug Discount Program http://www.cjaonline.net/events/SustSeries/Calls/Call20080918/OverviewSection340B2.pdf

[B18] Public Hospital Pharmacy Coalition Hospitals Struggle to Access Key Blood Products at Affordable Prices http://www.snhpa.org/public/documents/pdfs/IVIGPressReleaseandSummary.pdf

[B19] Dalakas MC High-dose intravenous immunoglobulin and serum viscosity: risk of precipitating thromboembolic events Neurology 1994 44 223 226 10.1212/WNL.44.2.223 8309562

[B20] Brannagan TH 3rd Intravenous gammaglobulin (IVIg) for treatment of CIDP and related immune-mediated neuropathies Neurology 2002 59 S33 S40 10.1212/WNL.59.12_suppl_6.S33 12499469

[B21] Stefanova E Pavlovic A Jovanovic Z Veselinovic N Despotovic I Stojkovic T Sternic N Kostic V Vascular risk factors in Alzheimer's disease—Preliminary report J Neurol Sci 2012 322 166 169 10.1016/j.jns.2012.07.065 22938734

[B22] Kalaria RN Akinyemi R Ihara M Does vascular pathology contribute to Alzheimer changes? J Neurol Sci 2012 322 141 147 10.1016/j.jns.2012.07.032 22884479

[B23] Rajabally YA Kearney DA Thromboembolic complications of intravenous immunoglobulin therapy in patients with neuropathy: a two-year study J Neurol Sci 2011 308 124 127 10.1016/j.jns.2011.05.035 21679973

[B24] Baxter Healthcare GAMMAGARD LIQUID [Immune Globulin Infusion (Human)] 10% http://www.baxter.com/products/biopharmaceuticals/downloads/gamliquid_PI.pdf

[B25] Haskin JA Warner DJ Blank DU Acute renal failure after large doses of intravenous immune globulin Ann Pharmacother 1999 33 800 803 10.1345/aph.18305 10466908

[B26] Zhang QL Koenig W Raum E Stegmaier C Brenner H Rothenbacher D Epidemiology of chronic kidney disease: results from a population of older adults in Germany Prev Med 2009 48 122 127 10.1016/j.ypmed.2008.10.026 19041887

[B27] Duhem C Dicato MA Ries F Side-effects of intravenous immune globulins Clin Exp Immunol 1994 97 79 83 8033440 PMC1550378

[B28] Bajaj NP Henderson N Bahl R Stott K Clifford-Jones RE Call for guidelines for monitoring renal function and haematological variables during intravenous infusion of immunoglobulin in neurological patients J Neurol Neurosurg Psychiatry 2001 71 562 563 10.1136/jnnp.71.4.562 11561055 PMC1763537

[B29] Brox AG Cournoyer D Sternbach M Spurll G Hemolytic anemia following intravenous gamma globulin administration Am J Med 1987 82 633 635 10.1016/0002-9343(87)90112-4 3826125

[B30] Frame WD Crawford RJ Thrombotic events after intravenous immunoglobulin Lancet 1986 2 468 2874460 10.1016/s0140-6736(86)92182-3

[B31] Pollreisz A Assinger A Hacker S Hoetzenecker K Schmid W Lang G Wolfsberger M Steinlechner B Bielek E Lalla E Klepetko W Volf I Ankersmit HJ Intravenous immunoglobulins induce CD32-mediated platelet aggregation in vitro Br J Dermatol 2008 159 578 584 10.1111/j.1365-2133.2008.08700.x 18565176

[B32] Kessary-Shoham H Levy Y Shoenfeld Y Lorber M Gershon H In vivo administration of intravenous immunoglobulin (IVIg) can lead to enhanced erythrocyte sequestration J Autoimmun 1999 13 129 135 10.1006/jaut.1999.0302 10441177

[B33] Matsuda M Hosoda W Sekijima Y Hoshi K Hashimoto T Itoh S Ikeda S Neutropenia as a complication of high-dose intravenous immunoglobulin therapy in adult patients with neuroimmunologic disorders Clin Neuropharmacol 2003 26 306 311 10.1097/00002826-200311000-00009 14646610

[B34] O'Nuallain B Acero L Williams AD Koeppen HP Weber A Schwarz HP Wall JS Weiss DT Solomon A Human plasma contains cross-reactive Abeta conformer-specific IgG antibodies Bioch 2008 47 12254 12256 10.1021/bi801767k 18956886

[B35] Szabo P Relkin N Weksler ME Natural human antibodies to amyloid beta peptide Autoimmun Rev 2008 7 415 420 10.1016/j.autrev.2008.03.007 18558354

[B36] Istrin G Bosis E Solomon B Intravenous immunoglobulin enhances the clearance of fibrillar amyloid-beta peptide J Neurosci Res 2006 84 434 443 10.1002/jnr.20886 16767774

[B37] Dodel R Balakrishnan K Keyvani K Deuster O Neff F Andrei-Selmer LC Röskam S Stüer C Al-Abed Y Noelker C Balzer-Geldsetzer M Oertel W Du Y Bacher M Naturally occurring autoantibodies against beta-amyloid: investigating their role in transgenic animal and in vitro models of Alzheimer's disease J Neurosci 2011 31 5847 5854 10.1523/JNEUROSCI.4401-10.2011 21490226 PMC6622820

[B38] Magga J Puli L Pihlaja R Kanninen K Neulamaa S Malm T Härtig W Grosche J Goldsteins G Tanila H Koistinaho J Koistinaho M Human intravenous immunoglobulin provides protection against Aβ toxicity by multiple mechanisms in a mouse model of Alzheimer's disease J Neuroinflammation 2010 7 90 10.1186/1742-2094-7-90 21138577 PMC3004875

[B39] Puli L Pomeshchik Y Olas K Malm T Koistinaho J Tanila H Effects of human intravenous immunoglobulin on amyloid pathology and neuroinflammation in a mouse model of Alzheimer's disease J Neuroinflammation 2012 9 105 10.1186/1742-2094-9-105 22642812 PMC3416679

[B40] Panza F Frisardi V Solfrizzi V Imbimbo BP Logroscino G Santamato A Greco A Seripa D Pilotto A Immunotherapy for Alzheimer's disease: from anti-β-amyloid to tau-based immunization strategies Immunotherapy 2012 4 213 238 10.2217/imt.11.170 22339463

[B41] Moreth J Mavoungou C Schindowski K Passive anti-amyloid immunotherapy in Alzheimer's disease: What are the most promising targets? Immun Ageing 2013 10 18 [Epub ahead of print] 10.1186/1742-4933-10-18 23663286 PMC3681567

[B42] Machimoto T Guerra G Burke G Fricker FJ Colona J Ruiz P Meier-Kriesche HU Scornik J Effect of IVIG administration on complement activation and HLA antibody levels Transpl Int 2010 23 1015 1022 10.1111/j.1432-2277.2010.01088.x 20412537

[B43] Abe Y Horiuchi A Miyake M Kimura S Anti-cytokine nature of natural human immunoglobulin: one possible mechanism of the clinical effect of intravenous immunoglobulin therapy Immunol Rev 1994 139 5 19 10.1111/j.1600-065X.1994.tb00854.x 7927413

[B44] Pigard N Elovaara I Kuusisto H Paalavuo R Dastidar P Zimmermann K Schwarz HP Reipert B Therapeutic activities of intravenous immunoglobulins in multiple sclerosis involve modulation of chemokine expression J Neuroimmunol 2009 209 114 120 10.1016/j.jneuroim.2009.01.014 19217671

[B45] Kessel A Ammuri H Peri R Pavlotzky ER Blank M Shoenfeld Y Toubi EJ Intravenous immunoglobulin therapy affects T regulatory cells by increasing their suppressive function J Immunol 2007 179 5571 5575 17911644 10.4049/jimmunol.179.8.5571

[B46] Samuelsson A Towers TL Ravetch JV Anti-inflammatory activity of IVIG mediated through the inhibitory Fc receptor Science 2001 291 484 486 10.1126/science.291.5503.484 11161202

[B47] Debré M Bonnet MC Fridman WH Carosella E Philippe N Reinert P Vilmer E Kaplan C Teillaud JL Griscelli C Infusion of Fc gamma fragments for treatment of children with acute immune thrombocytopenic purpura Lancet 1993 342 945 949 10.1016/0140-6736(93)92000-J 8105212

[B48] Nimmerjahn F Ravetch JV Anti-inflammatory actions of intravenous immunoglobulin Annu Rev Immunol 2008 26 513 533 10.1146/annurev.immunol.26.021607.090232 18370923

[B49] Kaneko Y Nimmerjahn F Ravetch JV Anti-inflammatory activity of immunoglobulin G resulting from Fc sialylation Science 2006 313 670 673 10.1126/science.1129594 16888140

[B50] Anthony RM Nimmerjahn F Ashline DJ Reinhold VN Paulson JC Ravetch JV Recapitulation of IVIG anti-inflammatory activity with a recombinant IgG Fc Science 2008 320 373 376 10.1126/science.1154315 18420934 PMC2409116

[B51] Anthony RM Wermeling F Karlsson MC Ravetch JV Identification of a receptor required for the anti-inflammatory activity of IVIG Proc Natl Acad Sci USA 2008 105 19571 19578 10.1073/pnas.0810163105 19036920 PMC2604916

[B52] Arriagada PV Growdon JH Hedley-Whyte ET Hyman BT Neurofibrillary tangles but not senile plaques parallel duration and severity of Alzheimer’s disease Neurology 1992 42 631 639 10.1212/WNL.42.3.631 1549228

[B53] Giannakopoulos P Herrmann FR Bussière T Bouras C Kövari E Perl DP Morrison JH Gold G Hof PR Tangle and neuron numbers, but not amyloid load, predict cognitive status in Alzheimer’s disease Neurol 2003 60 1495 1500 10.1212/01.WNL.0000063311.58879.01 12743238

[B54] Chai X Wu S Murray TK Kinley R Cella CV Sims H Buckner N Hanmer J Davies P O'Neill MJ Hutton ML Citron M Passive immunization with anti-tau antibodies in two transgenic models: reduction of tau pathology and delay of disease progression J Biol Chem 2011 286 34457 34467 10.1074/jbc.M111.229633 21841002 PMC3190817

[B55] Boutajangout A Ingadottir J Davies P Sigurdsson EM Passive immunization targeting pathological phospho-tau protein in a mouse model reduces functional decline and clears tau aggregates from the brain J Neurochem 2011 118 658 667 10.1111/j.1471-4159.2011.07337.x 21644996 PMC3366469

[B56] Singh R Barden A Mori T Beilin L Advanced glycation end-products: a review Diabetologia 2001 44 129 146 10.1007/s001250051591 11270668

[B57] Schmidt AM Sahagan B Nelson RB Selmer J Rothlein R Bell JM The role of RAGE in amyloid-beta peptide-mediated pathology in Alzheimer's disease Curr Opin Investig Drugs 2009 10 672 680 19579173

[B58] Deane R Du Yan S Submamaryan RK LaRue B Jovanovic S Hogg E Welch D Manness L Lin C Yu J Zhu H Ghiso J Frangione B Stern A Schmidt AM Armstrong DL Arnold B Liliensiek B Nawroth P Hofman F Kindy M Stern D Zlokovic B RAGE mediates amyloid-beta peptide transport across the blood–brain barrier and accumulation in brain Nat Med 2003 9 907 913 10.1038/nm890 12808450

[B59] Weber A Engelmaier A Teschner W Ehrlich HJ Schwarz HP Intravenous immunoglobulin (Vig) Gammagard liquid contains anti-RAGE IgG and sLRP Alzheimer’s Association 2009 International Conference on Alzheimer’s Disease 3 248

[B60] Shibata M Yamada S Kumar SR Calero M Bading J Frangione B Holtzman DM Miller CA Strickland DK Ghiso J Zlokovic BV Clearance of Alzheimer's amyloid-ss(1–40) peptide from brain by LDL receptor-related protein-1 at the blood–brain barrier J Clin Invest 2000 106 1489 1499 10.1172/JCI10498 11120756 PMC387254

[B61] Deane R Sagare A Zlokovic BV The role of the cell surface LRP and soluble LRP in blood–brain barrier Abeta clearance in Alzheimer's disease Curr Pharm Des 2008 14 1601 1605 10.2174/138161208784705487 18673201 PMC2895311

[B62] Sagare A Deane R Bell RD Johnson B Hamm K Pendu R Marky A Lenting PJ Wu Z Zarcone T Goate A Mayo K Perlmutter D Coma M Zhong Z Zlokovic BV Clearance of amyloid-beta by circulating lipoprotein receptors Nat Med 2007 13 1029 1031 10.1038/nm1635 17694066 PMC2936449

[B63] Mühl H Pfeilschifter J Anti-inflammatory properties of pro-inflammatory interferon-gamma Int Immunopharmacol 2003 3 1247 1255 10.1016/S1567-5769(03)00131-0 12890422

[B64] Lam L Whitsett CF McNicholl JM Hodge TW Hooper J Immunologically active proteins in intravenous immunoglobulin Lancet 1993 342 678 8103161 10.1016/0140-6736(93)91784-j

[B65] Grosse-Wilde H Blasczyk R Westhoff U Soluble HLA class I and class II concentrations in commercial immunoglobulin preparations Tissue Antigens 1992 39 74 77 10.1111/j.1399-0039.1992.tb01910.x 1574801

[B66] Blasczyk R Westhoff U Grosse-Wilde H Soluble CD4, CD8, and HLA molecules in commercial immunoglobulin preparations Lancet 1993 341 789 790 10.1016/0140-6736(93)90563-V 8096001

[B67] Claus R Werner H Schulze HA Walzel H Friemel H Are soluble monocyte-derived HLA class II molecules candidates for immunosuppressive activity? Immunol Lett 1990 26 203 210 10.1016/0165-2478(90)90147-I 2150835

[B68] Kekow J Reinhold D Pap T Ansorge S Intravenous immunoglobulins and transforming growth factor beta Lancet 1998 351 184 185 10.1016/S0140-6736(05)78212-X 9449877

[B69] van der Wal EA Gómez-Pinilla F Cotman CW Transforming growth factor-beta 1 is in plaques in Alzheimer and Down pathologies Neuroreport 1993 4 69 72 10.1097/00001756-199301000-00018 8453039

[B70] Wyss-Coray T Lin C Yan F Yu GQ Rohde M McConlogue L Masliah E Mucke L TGF-beta1 promotes microglial amyloid-beta clearance and reduces plaque burden in transgenic mice Nat Med 2001 7 612 618 10.1038/87945 11329064

[B71] Gelfand EW Differences between IGIV products: impact on clinical outcome Int Immunopharmacol 2006 6 592 599 10.1016/j.intimp.2005.11.003 16504921

[B72] Reipert BM Stellamor MT Poell M Ilas J Sasgary M Reipert S Zimmermann K Ehrlich H Schwarz HP Variation of anti-Fas antibodies in different lots of intravenous immunoglobulin Vox Sang 2008 94 334 341 10.1111/j.1423-0410.2008.001036.x 18266779

[B73] Mikolajczyk MG Concepcion NF Wang T Frazier D Golding B Frasch CE Scott DE Characterization of antibodies to capsular polysaccharide antigens of Haemophilus influenzae type b and Streptococcus pneumoniae in human immune globulin intravenous preparations Clin Diagn Lab Immunol 2004 11 1158 1164 15539522 10.1128/CDLI.11.6.1158-1164.2004 PMC524781

[B74] Patrias LM Klaver AC Coffey MP Loeffler DA Specific antibodies to soluble alpha-synuclein conformations in intravenous immunoglobulin preparations Clin Exp Immunol 2010 161 527 535 10.1111/j.1365-2249.2010.04214.x 20646004 PMC2962971

[B75] Dhainaut F Guillaumat PO Dib H Perret G Sauger A de Coupade C Beaudet M Elzaabi M Mouthon L In vitro and in vivo properties differ among liquid intravenous immunoglobulin preparations Vox Sang 2013 104 115 126 10.1111/j.1423-0410.2012.01648.x 23003576 PMC3580880

[B76] Manlhiot C Yeung RS Chahal N McCrindle BW Intravenous immunoglobulin preparation type: association with outcomes for patients with acute Kawasaki disease Pediatr Allergy Immunol 2010 21 515 521 20546528 10.1111/j.1399-3038.2010.00987.x

[B77] Roifman CM Schroeder H Berger M Sorensen R Ballow M Buckley RH Gewurz A Korenblat P Sussman G Lemm G Comparison of the efficacy of IGIV-C, 10% (caprylate/chromatography) and IGIV-SD, 10% as replacement therapy in primary immune deficiency. A randomized double-blind trial Int Immunopharmacol 2003 3 1325 1333 10.1016/S1567-5769(03)00134-6 12890430

[B78] Crow AR Lazarus AH The mechanisms of action of intravenous immunoglobulin and polyclonal anti-d immunoglobulin in the amelioration of immune thrombocytopenic purpura: what do we really know? Transfus Med Rev 2008 22 103 1016 10.1016/j.tmrv.2007.12.001 18353251

[B79] Becker T Küenzlen E Salama A Mertens R Kiefel V Weiss H Lampert F Gaedicke G Mueller-Eckhardt C Treatment of childhood idiopathic thrombocytopenic purpura with Rhesus antibodies (anti-D) Eur J Pediatr 1986 145 166 169 10.1007/BF00446055 3021462

[B80] Salama A Kiefel V Mueller-Eckhardt C Effect of IgG anti-Rho(D) in adult patients with chronic autoimmune thrombocytopenia Am J Hematol 1986 22 241 250 10.1002/ajh.2830220304 3087158

[B81] Svetlicky N Ortega-Hernandez OD Mouthon L Guillevin L Thiesen HJ Altman A Kravitz MS Blank M Shoenfeld Y The advantage of specific intravenous immunoglobulin (sIVIG) on regular IVIG: experience of the last decade J Clin Immunol 2013 33 Suppl 1 S27 S32 23229779 10.1007/s10875-012-9842-5

[B82] Ryan DA Mastrangelo MA Narrow WC Sullivan MA Federoff HJ Bowers WJ Abeta-directed single-chain antibody delivery via a serotype-1 AAV vector improves learning behavior and pathology in Alzheimer's disease mice Mol Ther 2010 18 1471 1481 10.1038/mt.2010.111 20551911 PMC2927061

[B83] Käsermann F Boerema DJ Rüegsegger M Hofmann A Wymann S Zuercher AW Miescher S Analysis and functional consequences of increased Fab-sialylation of intravenous immunoglobulin (IVIG) after lectin fractionation PLoS One 2012 7 e37243 10.1371/journal.pone.0037243 22675478 PMC3366990

[B84] McGeer PL Akiyama H Itagaki S McGeer EG Activation of the classical complement pathway in brain tissue of Alzheimer patients Neurosci Lett 1989 107 341 346 10.1016/0304-3940(89)90843-4 2559373

[B85] Webster S Lue L-F Brachova L Tenner AJ McGeer PL Terai K Walker DG Bradt B Cooper NR Rogers J Molecular and cellular characterization of the membrane attack complex, C5b-9, in Alzheimer's disease Neurobiol Aging 1997 18 415 421 10.1016/S0197-4580(97)00042-0 9330973

[B86] Loeffler DA Using animal models to determine the significance of complement activation in Alzheimer's disease J Neuroinflammation 2004 1 18 10.1186/1742-2094-1-18 15479474 PMC529311

[B87] Rossi F Dietrich G Kazatchkine MD Anti-idiotypes against autoantibodies in normal immunoglobulins: evidence for network regulation of human autoimmune responses Immunol Rev 1989 110 135 149 10.1111/j.1600-065X.1989.tb00031.x 2676846

